# Spermatogonial Stem Cells (SSCs) in Buffalo (*Bubalus bubalis*) Testis

**DOI:** 10.1371/journal.pone.0036020

**Published:** 2012-04-20

**Authors:** Ranjeet Singh Mahla, Niranjan Reddy, Sandeep Goel

**Affiliations:** Laboratory for the Conservation of Endangered Species, Centre for Cellular and Molecular Biology, Council for Scientific and Industrial Research, Hyderabad, India; National Cancer Institute, United States of America

## Abstract

**Background:**

Water buffalo is an economically important livestock species and about half of its total world population exists in India. Development of stem cell technology in buffalo can find application in targeted genetic modification of this species. Testis has emerged as a source of pluripotent stem cells in mice and human; however, not much information is available in buffalo.

**Objectives and Methods:**

Pou5f1 (Oct 3/4) is a transcription factor expressed by pluripotent stem cells. Therefore, in the present study, expression of POU5F1 transcript and protein was examined in testes of both young and adult buffaloes by semi-quantitative reverse transcriptase-polymerase chain reaction (RT-PCR) and immunohistochemical analysis. Further, using the testis transplantation assay, a functional assay for spermatogonial stem cells (SSCs), stem cell potential of gonocytes/spermatogonia isolated from prepubertal buffalo testis was also determined.

**Results:**

Expression of POU5F1transcript and protein was detected in prepubertal and adult buffalo testes. Western blot analysis revealed that the POU5F1 protein in the buffalo testis exists in two isoforms; large (∼47 kDa) and small (∼21 kDa). Immunohistochemical analysis revealed that POU5F1 expression in prepubertal buffalo testis was present in gonocytes/spermatogonia and absent from somatic cells. In the adult testis, POU5F1 expression was present primarily in post-meiotic germ cells such as round spermatids, weakly in spermatogonia and spermatocytes, and absent from elongated spermatids. POU5F1 protein expression was seen both in cytoplasm and nuclei of the stained germ cells. Stem cell potential of prepubertal buffalo gonocytes/spermatogonia was confirmed by the presence of colonized DBA-stained cells in the basal membrane of seminiferous tubules of xenotransplanted mice testis.

**Conclusion/Significance:**

These findings strongly indicate that gonocytes/spermatogonia, isolated for prepubertal buffalo testis can be a potential target for establishing a germ stem cell line that would enable genetic modification of buffaloes.

## Introduction

Water buffalo (*Bubalus bubalis*) is an inhabitant of the Asian continent and about half of its total world population exists in India. Approximately 140 million tons of buffalo milk is estimated to be produced annually in India, thereby making buffalo an important livestock species here [1]. Selective breeding and improved management have made positive impact on buffalo production worldwide, however, buffalo has been regarded as a sluggish breeder with low reproductive efficiency, characterized by delayed puberty, seasonality, anoestrus, low conception rate and long calving intervals [2]. Several reproductive technologies from bovine have been adapted for improvement of buffalo production, albeit with various limitations. Therefore, there is a need to explore newer areas that can find application in genetic improvement of buffalo more efficiently. One such area is male germline stem cell technology that still remains unexplored in this species.

Male germline stem cells have received a great deal of attention in recent years, as it is possible to isolate and culture them *in vitro* [3]–[9]. Genetic changes can be induced in these cells, to alter their culture conditions thereby altering their epigenetic status [10].Male germline stem cells can be genetically modified and they further differentiate to spermatozoa, following transplantation into a recipient testis eventually producing transgenic offspring [11]–[16]. A comprehensive knowledge of male germline stem cell biology will help us better understand the stem cell regulation in testis and eventually enhance our understanding of male fertility and stem cell biology in human and other model organisms [17], [18]. In the case of livestock species, where no embryonic stem (ES) cell line with germ-line characteristics has been reported to date, a male germ stem cell line could be used to produce transgenic animals in a way that overcomes the numerous shortcomings of the conventional pronuclear injection method. If these cell lines are established, the homologous recombination technique could be applied to create targeted mutated animals. Creation of such animals is presently achieved inefficiently by somatic cell nuclear transfer (SCNT) technique [19]–[22]. However, no male germ stem cell line has yet been established for a livestock species; most likely reason being the lack of sufficient understanding of expression of vital pluripotency-related markers in male germ stem cells.

Examination of pluripotency cell-specific proteins in the testis can provide useful information regarding the stem cell capabilities of germ cells. Pou5f1 (also known as Oct3/4), a POU and homeobox transcription factor, is essential for maintaining the pluripotential phenotype [23]. It is expressed in pluripotent cells of morula, cells of the inner cell mass (ICM), epiblasts, and primordial germ cells (PGCs) [24]. In female PGCs, Pou5f1 is repressed by the onset of meiotic prophase I (E13-14) and is re-expressed after birth, coinciding with the growth phase of oocytes. In male embryos, Pou5f1 expression persists in germ cells throughout fetal development. After birth, it is maintained in proliferating gonocytes, pro-spermatogonia and later in undifferentiated spermatogonia [25], [26]. In addition, ES, embryonic germ (EG), embryonic carcinoma (EC) cells, the epiblast and PGCs, respectively, also express Pou5f1 as long as they remain undifferentiated [27]–[29]. Expression of POU5F1 has been reported in testes of developing pig [30], cattle [31], marmoset (*Callithrix jacchus*) [32] and humans [32]. A recent report describes expression of POU5F1 in the testis of an endangered bovid; the Indian black buck (*Antelope cervicapra L*) [33]. This report also confirmed stem cell potential of black buck spermatogonia by the testis transplantation technique.

The testis transplantation technique is an assay for detecting the presence of spermatogonial stem cells in a cell population [34]. Complete spermatogenesis following cross-species (xenogenic) germ cell transplantation in mice testis is accounted for in rat [35] and hamster [36]. Germ cell transplantation from genetically distant donor species, including farm animals, into mice resulted only in colonization or proliferation of SSCs, but not in complete spermatogenesis [37]–[40]. Although germ cell transplantation from non-rodent species into mouse testis did not result in complete spermatogenesis, till date it is the only available bioassay for detecting the stem cell potential of germ cells in a given population of donor testis cells from any species [37], [38]. The stem cell potential of buffalo gonocyte/spermatogonia still remains elusive.

Identification of male germline cell-specific markers help in distinguishing these cells from other cell types in the testis. These markers have been especially useful in identification of male germ cells while isolation and purification from the testis [41], [42]. Identification of a species-specific marker can aid in tracing spermatogonia of the donor species in xenotransplanted mice testis [30], [33], [43]. Recently, we reported lectin DBA as a specific marker for spermatogonia in testes of prepubertal buffalo, and utilized it for their characterization in short-term culture [41]. However, stem cell potential of buffalo spermatogonia has not yet been determined. The objective of the present study was to examine the expression of POU5F1 transcript and protein in prepubertal and adult buffalo testes. Further, stem cell potential of buffalo gonocytes/spermatogonia, isolated from prepubertal testis, was determined using the testis xenotransplantation assay.

## Results

**Figure 1:**
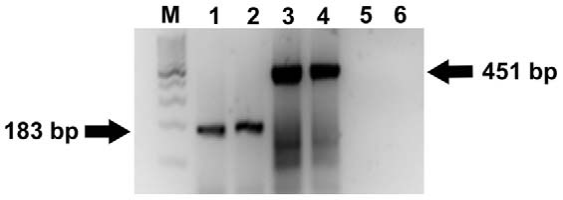
Detection of *POU5F1* transcript in buffalo testis. Total RNA was extracted from prepubertal (4- to 6- month old; lanes 1, 3, 5) and from adult (2- to 3- year old; lanes 2, 4, 6) buffalo testes and pooled for template. Transcript of *POU5F1* was detected both in prepubertal (lane 1) and adult (lane 2) testes. *BETA-ACTIN* was used for normalizing RNA samples in prepubertal (lanes 3) and adult (lane 4) testes. Isolated RNA without reverse transcription to check genomic DNA contamination in prepubertal (lane5) and adult (lane 6) testes. M: 100 base pair molecular weight marker.

Semi-quantitative reverse transcription-polymerase chain reaction (RT-PCR) analysis showed expression of *POU5F1* mRNA in testes of prepubertal and adult buffaloes (Fig. 1). PCR product size of 183 bp for *POU5F1* primers was observed, which was expected. The expression level of *POU5F1* mRNA was similar in both prepubertal and adult testes.

**Figure 2:**
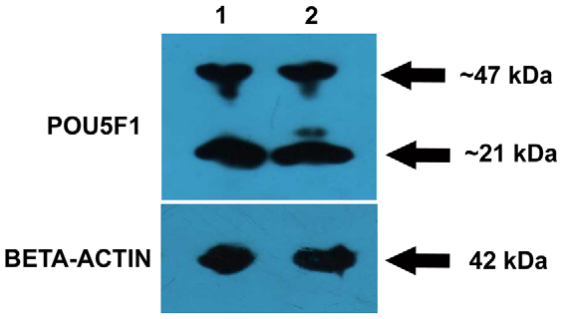
Western blot analysis to demonstrate that anti-POU5F1 antibody identifies proteins of defined molecular weight in buffalo testes. Protein lysates from prepubertal (4- to 6- month old; lane 1) and from adult (2- to 3- year old; lane 2) buffalo testes shows that POU5F1 protein exists in two isoforms; large (∼47 kDa) and small (∼21 kDa). BETA-ACTIN was used for normalizing protein samples.

Anti-Pou5f1 antibody was able to identify proteins of definite sizes in the buffalo-testes lysates as shown by the Western blot analysis (Fig. 2). The anti-Pou5f1 antibody identified two isoforms of POU5F1 protein in testes of both prepubertal and adult buffalo, a large fragment of approximately 47 kDa and a smaller fragment of approximately 21 kDa in the immunoblots.

**Figure 3:**
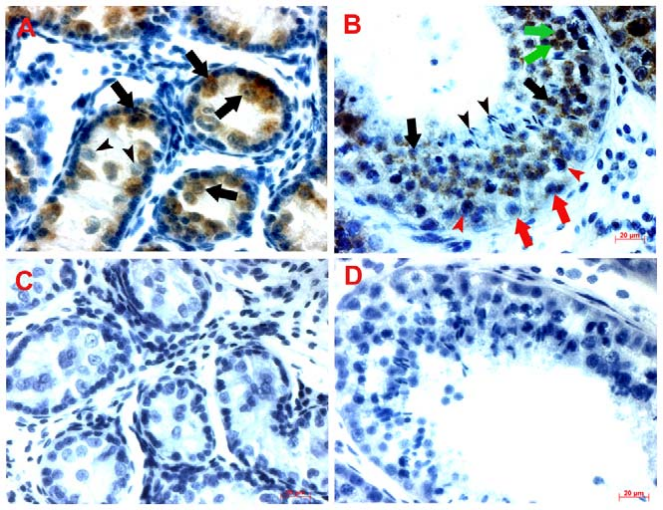
Immunohistochemical analysis of POU5F1 protein in prepubertal and adult testis sections. (A) In 4-month-old testis section, most germ cells express POU5F1 (black arrows) and a few germ cells did not stain (arrowheads). POU5F1 expression is not seen in Sertoli and interstitial cells. (B) In 2-year-old testis section, POU5F1 expression is present in cytoplasm (black arrows) and nuclei of (green arrows) of round spermatids. At this age, spermatocytes (red arrowheads) and spermatogonia (red arrows) show very weak cytoplasmic localization of POU5F1 protein. Elongated spermatids show no expression of POU5F1 protein (black arrowheads). In a negative control (C, D), where primary antibody was omitted, no positive cells are present. Scale bar = 20 µm.

POU5F1 expression in the prepubertal buffalo testes was present in the germ cells, which were easily distinguishable by their large size, topological position and morphology (Fig. 3A). However, POU5F1 expression was not detected in somatic cells such as Sertoli cells and interstitial cells (Fig. 3A). POU5F1 expression was observed both in the cytoplasm and in the nuclei of the stained germ cells. In a few spermatogonia, POU5F1 expression was weak or absent (Fig. 3A). In adult testes, POU5F1 expression was weak in spermatogonia and spermatocytes, but strong in post-meiotic germ cells, such as round spermatids (Fig. 3B). At this stage, POU5F1 expression was not detected in elongated spermatids. POU5F1 expression was present mostly in the cytoplasm of differentiated germ cells, however, a few germ cells showed nuclear localization of POU5F1 at the adult stage. No positive staining of POU5F1 was seen in negative control testes sections (Fig. 3C, D).

**Figure 4:**
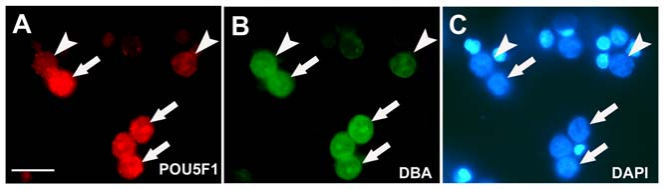
Fluorescence-double staining of testicular cells. Cells isolated from a 5-month-old buffalo testis stained with POU5F1 (A), and DBA (B). Most DBA binding cells are positive for POU5F1 expression (arrows). POU5F1 expression is occasionally weak or absent in DBA-positive cells (arrowheads). (C) Cells stained with DAPI. Scale bar = 50 µm.

Cells isolated from prepubertal buffalo testes following two-step-enzymatic digestion contained 14.5±1.3% DBA- and 11.5±2.3% POU5F1-positive gonocytes/spermatogonia. The percentage of DBA-positive cells did not differ significantly from the POU5F1-positive cell (P<0.05). Double-immunofluroscence analysis of isolated testicular cells revealed that POU5F1 expression was co-localized in most DBA-positive gonocytes/spermatogonia (Fig. 4A–C). However, occasionally in a few DBA-positive cells, POU5F1 expression was weak or absent.

**Figure 5:**
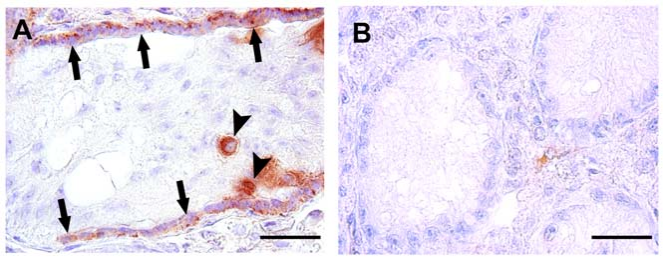
Analysis of xenotransplanted mice testes one-month after transplantation using a buffalo gonocytes/spermatogonia-specific marker, *Dolichos biflorus* agglutinin (DBA). (A) DBA stained gonocytes/spermatogonia (arrows) that colonized the basal membrane of the recipient testis. Note that a few uncolonized DBA-positive gonocytes/spermatogonia are seen in lumen of seminiferous tubules (arrowheads). (B) No DBA-positive cells are visible in the contralateral testis section. Scale bar = 50 µm.

To determine the stem cell potential of gonocytes/spermatogonia, testicular cells from prepubertal buffalo testes were transplanted into the testes of immunodeficient mice. One month after transplantation, buffalo gonocytes/spermatogonia were detected in the basement membrane of seminiferous tubules of all recipient mice testes, as determined by DBA staining (Fig. 5A). At this time, DBA-stained buffalo gonocytes/spermatogonia were found in 8.5±3.2% of tubule/sections. The injected spermatogonia migrated to the basement membrane of the seminiferous tubule and appeared as chain of cells that were stained with DBA. These cells were located in the area of the seminiferous tubule, consistent with the stem cell niche. Occasionally, a few DBA-positive cells were seen in the lumen of seminiferous tubules. DBA-positive cells were not found in the contralateral testis, used as control (Fig. 5B).

## Discussion

Pou5f1 is a transcription factor required to maintain the pluripotency and self-renewal of ES cells [44], [45]. Pou5fl controls a cascade of pathways that are intricately connected to govern pluripotency, self-renewal, genome surveillance and cell fate determination [46]. To investigate the stem cell characteristics of germ cells in buffalo, we examined the expression of Pou5f1, both at transcript and protein level in prepubertal and adult testes. In the present study, expression of *POU5F1* transcript was detected both in prepubertal and adult buffalo testis. In a recent study, expression of *POU5F1* transcript was reported in buffalo embryonic stem cell (ESC) like cells [47]. However in the same study, it was reported that *POU5F1* is expressed as four pseudogenes. The *POU5F1* primers used in this study amplified a PCR product of a definite size in buffalo testes. It is likely that the primers designed in the present study were in a conserved region of buffalo *POU5F1* gene. The possibility of existence of a single transcript for the *POU5F1* gene in buffalo testis cannot be ruled out.

Western blot analysis showed that the anti-POU5F1 antibody identifies two isoforms of POU5F1 protein buffalo testes. The smaller fragment in POU5F1 immunoblots indicates towards the cytoplasmic isoform of POU5F1 protein in buffalo testis. A similar finding for NANOG protein was reported in pig testis, where a truncated protein of smaller size was observed [30]. In a recent report, Western blot of POU5F1 revealed presence of four distinct visible bands in buffalo ESC-like cells, with the upper most band (∼38 kD) corresponding to the parent POU5F1 and the other three proteins (size ranging from 35.5 kDa to <24 kDa) resulting from pseudogene expression, leading to the production of truncated proteins [47]. In the present study, the uppermost band of POU5F1 protein was of much larger size (i.e., ∼47 kDA). This discrepancy could be due to the difference in the size of POU5F1 protein expressed in testis of buffalo. This could also be attributed to the different antibody used in this study which identifies larger isoform of POU5F1 protein.

In this study, we showed that gonocytes/spermatogonia specifically expressed POU5F1 in the prepubertal buffalo testis until the gonocytes/spermatogonia migrated to the basal membrane. This finding is in agreement with our earlier report where we showed expression of POU5F1 protein in gonocytes/spermatogonia of prepubertal buffalo testis [41]. Similarly, in neonatal cattle testis [31], and in fetal and early postnatal marmoset (*Callithrix jacchus*) and human testes [32], gonocytes/spermatogonia express POU5F1. However, the expression of POU5F1 protein in gonocytes/spermatogonia of prepubertal buffalo testis is in disagreement with finding in neonatal pig where, POU5F1 expression was absent until first three weeks after birth, both at transcript and protein level [30]. Similarly in prepubertal and adult human testes, POU5F1 was not detected in any of the germ cells [48]. In the present study, the expression of POU5F1 protein was seen both in the nuclei and in the cytoplasm of the stained germ cells in prepubertal testis. In mouse testes, POU5F1 expression has a predominant nuclear localization in undifferentiated germ cells such as PGCs, gonocytes, and spermatogonia [49]. Upon commitment of SSCs to differentiate, POU5F1 protein becomes localized in the cytoplasm and the expression levels fall with the complete absence of expression by the onset of meiosis [25]. Nevertheless, our finding that POU5F1 is expressed both in the nuclei and cytoplasm of germ cells in prepubertal testis is suggestive of their stem cell potential. Further, expression of POU5F1 in the round spermatids suggests that this protein may have some role in the meiotic stage of germ cells, which needs to be determined. In conclusion, POU5F1 expression in the buffalo testis is rather dynamic and is observed both in undifferentiated and differentiated germ cells.

Our finding that POU5F1 expression was markedly upregulated in the gonocytes/spermatogonia but was weak or absent from a few spermatogonia, raised doubts about stem cell potential of gonocytes/spermatogonia. The spermatogonial stem cell transplantation technique is an assay for detecting the presence of spermatogonial stem cells in a population of cells [34]. Since most of the POU5F1 expressing gonocytes/spermatogonia showed affinity for lectin-DBA, the testes of the recipient mice were examined for the presence of buffalo gonocytes/spermatogonia using DBA staining. Lectin DBA is a specific marker of buffalo spermatogonia [41] and shows no affinity for mouse testicular cells [30], [33], [50]. Using testis transplantation assay, we found that gonocytes/spermatogonia from the testis of prepubertal buffalo could colonize recipient testes. One month after transplantation, DBA-positive germ cells were detected in the testes of the recipient mice located in the area of the seminiferous tubule, consistent with the stem cell niche. The buffalo gonocytes/spermatogonia could not only colonize the recipient mice testis but also showed lateral expansion in the seminiferous tubules of mice. The chain of cells connected by intercellular bridges represents proliferating germ cells in the testes of the xenotransplanted recipient mice [33], [37], [38]. In the present study, enrichment of gonocyte/spermatogonia population was not done. This explains the small number of seminiferous tubules colonized with buffalo gonocytes/spermatogonia in xenotransplanted mice testis. Proliferation and differentiation of xenogenic germ cells depends on the microenvironment of the seminiferous tubule and interactions between germ cells and somatic cells [51]. It is likely that the microenvironment in the mice testis supports proliferation of buffalo gonocyte/spermatogonia. Similarly, spermatogonia from large domestic animals such as boars, bulls, and stallions in the prepubertal stage, have shown colonization and proliferative ability in recipient mice testis [38]. In the transplanted testis, occasionally, a few DBA-positive cells were present in the lumen of seminiferous tubules. This raises the possibility that not all gonocytes/spermatogonia were able to migrate to the basement membrane of the seminiferous tubule, the area consistent with the stem cell niche, to colonize the recipient testis due to lack of stem cell potential. This is in agreement with the finding in the present study where a few DBA-positive cells showed weak or no POU5F1 expression in double immunoflouroscence analysis of isolated testicular cells from prepubertal buffaloes. These findings suggest that non-colonized DBA-positive cells represent gonocytes/spermatogonia that have weak or no expression of POU5F1. This, however, could not be confirmed in xenotransplanted testis as mice spermatogonia express Pou5f1 [49]. Alternatively, early collection of recipient testis (1-month-post-transplantation) may not have given sufficient time for gonocyte/spermatogonia to colonize the recipient testis. Further studies in this direction would help in better understanding.

In conclusion, pluripotent-cell specific marker POU5F1 is expressed in buffalo testes, both at prepubertal and adult stages. Colonization of gonocytes/spermatogonia in xenotransplanted recipient mice testis confirms the presence of SSC population in testis of prepubertal buffalo for the first time. Findings from this study can be used for initiating the use of SSC-based technologies for the fertility restoration and genetic modification of buffalo.

## Materials and Methods

### Ethics Statement

All animal experiments were approved by the Institutional Animal Ethics Committee of the Centre for Cellular and Molecular Biology, Hyderabad, India.

### Collection of Buffalo Testes

The testes from prepubertal (aged 4–6 months; n = 5) and adult (aged 2–3 years; n = 4) water buffaloes (*Bubalus bubalis*) of Murrah breed were collected from Municipal Slaughterhouse, Hyderabad, India. A small piece of testis tissue was submerged immediately after collection in RNALater® (Ambion, Inc; www.ambion.com) following manufacturer's instructions and stored at −20°C until isolation of RNA. For histochemical analysis, testes tissue was immediately fixed in Bouin's fixative following collection. For protein isolation, a small piece of testes tissue was immersed in dissolving buffer (7 M urea, 2 M thiourea, 4% CHAPS, 18 mM Tris-HCl, 14 mM Tris-Base, 2 tablets EDTA protease inhibitor, 0.2% Triton-X, 50 mM DTT) and stored at −20°C until isolation of protein. For testicular cell isolation, the testes from prepubertal buffaloes (n = 3) were transported in DMEM/F12 containing 15 mM HEPES (DMEM/F12-HEPES; Invitrogen, www.invitrogen.com) on ice within 1–2 hour to the laboratory.

### Isolation of RNA and Reverse Transcription-Polymerase Chain Reaction (RT-PCR) Analysis

Total RNA was prepared from testes of prepubertal and adult buffaloes. The stored testes tissue pieces were removed from RNALater® and processed for RNA isolation using TRIZOL reagent (Invitrogen; www.invitrogen.com) according to the manufacturer's instructions. Extracted RNAs were diluted with DEPC-water and incubated with 10 units of RNase free-DNase (Roche, www.roche.com) for 30 min at room temperature. Incubating the samples at 70°C for 15 min inhibited DNase activity and samples were stored on ice. Random Primers and RNase OUT (both from Invitrogen) were added to the RNA solution, incubated for 5 min at 65°C and set on ice. For reverse transcription, MMLV high performance reverse transcriptase (Epicentre biotechnologies, www.epibio.com) was added to the RNA solution and incubated for 10 min at 25°C, for 60 min at 37°C and for 5 min at 90°C (RT +). At the same time, the reactions without the addition of reverse transcriptase enzyme were done to check genomic DNA contamination (RT −). PCR amplification was carried out on 1 µl of the cDNA per 19 µl of PCR reaction mixture containing 2 mM MgCl_2_, 0.25 mM dNTPs, 1× PCR buffer, 5 pmol of each primers and 1 U of *Taq* DNA polymerase (AmpliTaq Gold™, Applied Biosystems, www.appliedbiosystems.com). The following primers were used for amplification of specific genes: *POU5F1* 5′- GTTTTGAGGCTTTGCAGCTC -3′, 5′- TCTCCAGGTTGCCTCTCACT-3′, 183 base pair (bp) (GeneBank access. no. GU997625.1) (annealing at 58°C, 30 cycles); *β-ACTIN* 5′-CGATCCACACAGAGTACTTGCG-3′, 5′-CGAGCGTGGCTACAGTTCACC-3′, 451 bp (GeneBank access. no. NM_001101) (annealing at 58°C, 30 cycles). The PCR products were separated and visualized by 2% agarose gel electrophoresis containing 0.5 µg/ml ethidium bromide. All PCR products were sequenced to confirm identity.

### Western-Blot Analysis

To demonstrate that anti-POU5F1 antibody, which was raised against mouse antigens, is able to recognize proteins of predicted molecular weight in buffalo testis, Western blot analysis was performed. Total proteins from buffalo testes were extracted upon homogenization by sonication in a dissolving buffer. Lysed samples (20 µg) were subjected to electrophoresis in 12% SDS-polyacrylamide gel. The gels were transferred onto the polyvinylidene difluoride membrane (Millipore, www.millipore.com). The membranes were blocked with Blocker™ casein in PBS (Thermo Scientific; www.piercenet.com) for 1 h at room temperature. The blocked membranes were incubated with polyclonal rabbit anti-Pou5f1 (previously known as Oct3/4) (1∶5000; Millipore, www.millipore.com) or monoclonal mouse anti-beta actin (1∶5000; Sigma-Aldrich; www.sigmaaldrich.com) in Blocker™ casein in PBS overnight at 4°C. The membranes were then washed with PBS-T (136 mM NaCl, 2.68 mM KCl, 8.1 mM Na_2_HPO_4_, 1.47 mM KH_2_PO_4_, and 0.1% (v/v) Tween-20) and incubated with goat anti-rabbit or goat anti-mouse HRP-conjugated secondary antibody (1∶10000; both from Calbiochem, www.calbiochem.com) in PBS-T for 1 h at room temperature. The blots were visualized using the Immobilon Western chemiluminescence HRP substrate system (Millipore) following exposure to X-ray film (Amersham, www.gelifesciences.com).

### Histochemistry

Fixed testicular tissues from prepubertal and adult buffalo testes were dehydrated, embedded in paraffin and sectioned (6 µm thick). Dilutions of primary and secondary antibodies were done in PBS with 1% BSA (Sigma, www.sigmaaldrich.com). Briefly, sections were dewaxed, rehydrated and blocked with 10% fetal bovine serum (Gibco, www.invitrogen.com) in PBS for 30 min, incubated with anti-Pou5f1 antibody (1∶200) overnight at 4°C, washed several times with PBS, incubated with 3% H_2_O_2_ (Fisher Scientific, www.fishersci.com) for 10 min, washed three times with PBS, incubated with corresponding HRP-conjugated secondary antibody, i.e., goat anti-rabbit IgG (Calbiochem; 1∶300) for 30 min at 37°C, rinsed three times with PBS, incubated for 3–5 min in DAB substrate kit (Vector Laboratories, www.vectorlabs.com) according to the manufacture's instructions, rinsed thoroughly in distilled water, counterstained with hematoxylin, dehydrated and mounted in Vectamount (Vector Laboratories) and observed under a Zeiss Axioplan 2 microscope (Carl Zeiss AG, Gottingen, Germany). In negative controls, primary antibody was omitted and instead the section was incubated with 1% BSA in PBS.

### Preparation of Testicular Cell Suspension and Immunofluorescence Analysis

All chemicals were from Sigma (www.sigmaaldrich.com) unless mentioned otherwise. Testicular cell suspension was prepared from prepubertal buffalo testes as described previously [41] with some modifications. Briefly, after washing several times with PBS, tunica and other visible connective tissues were removed. The testes were minced with scissors and incubated in DMEM/F12-HEPES medium supplemented, 100 IU/ml-50 µg/ml penicillin-streptomycin, 40-mg/ml gentamycin, 1.5 mg/ml collagenase type IV and 5 µg/ml DNase at 35°C for 30 min in a shaking water bath operated at 100 cycles/min. The digested testis was washed three times with DMEM/F12-HEPES medium to remove most of the interstitial cells surrounding the seminiferous tubules. The resultant tubule fragments were incubated in second digestion mix that contained DMEM/12-HEPES medium, 1.5 mg/ml trypsin and 5 µg/ml DNase for 30 min in the conditions described above. The dispersed cells were washed twice with medium, suspended in DMEM/F12 with 10% FBS (Invitrogen) and filtered through 100-µm and 40-µm cell strainer (BD Falcon, www.bdbiosciences.com) successively. The cells were collected by centrifugation at 600 g, washed twice with DMEM/F12-HEPES. A small quantity of cells were collected and processed for immunofluorscence analysis. The remaining cells were resuspended in DMEM/F12-HEPES containing 10% FBS and 2 µg/ml DNase and stored on ice until injection. The viability of cells was greater than 85% as determined by trypan-blue dye exclusion.

Testicular cells isolated from prepubertal buffalo testes were analysed for the presence of lectin-*Dolichos biflorus* agglutinin (DBA)-positive cells, a buffalo gonocyte/spermatogonia specific marker [41]. Simultaneously, cells were stained with anti-POU5F1 antibody to determine POU5F1 protein expression in DBA-binding cells. Briefly, cells were suspended and centrifuged onto poly-l-lysine coated coverslips. The cells were fixed in Bouin's fixative for 20 min. After washing with PBS, the fixed cells were permeabilized with 0.2% Triton X-100 in PBS for 10 min and blocked for 1 hr at room temperature or overnight at 4°C with PBS containing 10% goat serum, 5% bovine serum albumin (BSA) and 0.1% Triton X-100. Incubation of cells with the primary antibody was carried out for 1 hr at room temperature or overnight at 4°C in PBS containing 1% goat serum, 0.1% Triton X-100. Following overnight incubation in an antibody at the given dilution (1∶200), cells were washed several times with PBS, incubated with goat anti-rabbit-Cy3 (Molecular probes; www.invitrogen.com; 1∶200) and DBA-FITC (Vectors Laboratories; 1∶100) for 1 h at 37°C, rinsed three times with PBS, stained with 100 ng/mL DAPI (Molecular Probes) for 30 min, mounted in slow-Fade (Molecular Probes) and observed under an Axioplan 2 microscope fitted with an epifluorescent lamp. In negative controls, primary antibody and lectin were omitted and instead the sections were incubated with 1% BSA in PBS. Approximately 500 cells in each trial (n = 3) were counted in random fields to evaluate the DBA- and POU5F1-positive cells. The results are presented as mean ± S.E.M. The statistical analysis was conducted using ANOVA. Differences were determined by analyzing the data with Fisher's PSLD test for significance between the means. The level of significance was set at P<0.05.

### Donor Cell Transplantation and Analysis of the Recipient Testis

Balb/c nude (nu/nu) mice 8–10-weeks (n = 6) of age were used as recipient animals to avoid immunological reaction of donor cells. The mice were kept under specific pathogen-free conditions and food, water and bedding were autoclaved before use. The mice were housed in 12 hr light and 12 hr dark cycle at constant temperature and provided with food and water ad libitum. At least 4 weeks before donor cell transplantation, the mice were injected busulfan (35 mg/kg body weight [BW]; Sigma) intraperitoneally to deplete endogenous germ cells in the testes [31]. The recipient mice were anesthetized by intraperitoneal administration of ketamine (0.1 mg/kg BW) and xylazine (0.5 mg/kg BW) in sterile physiological saline. The testes were exposed through a midline abdominal incision, and approximately 10 to 15 µl of donor cell suspension containing 50–60×10^6^ cells/ml was injected through the efferent duct as described previously [52]. Trypan blue (0.03%) was added to the injection media that allowed visualization of a successful injection to the seminiferous tubules. Approximately 70 to 80% of the surface tubules were filled with each injection. The contralateral testis was used as the negative control.

One month after donor-cell transplantation, the recipient mice were killed by CO_2_ inhalation, and both testes were removed. Testes were fixed and processed for the histochemical analysis to detect the presence of buffalo spermatogonia/gonocyte using lectin- *Dolichos biflorus* agglutinin (DBA) staining [41]. Briefly, a testis section was incubated with 3% H_2_O_2_ for 10 min, washed with PBS, incubated in 5% BSA in PBS for 15 min, incubated with DBA-conjugated horseradish peroxidase (DBA-HRP; E.Y. Laboratories, www.eylabs.com; 1∶100) for 1 hr at 37°C in a moist chamber, rinsed three times with PBS, incubated for 3–5 min in DAB substrate kit, rinsed thoroughly in distilled water, counterstained with hematoxylin, dehydrated, mounted in Vectamount (Vector Laboratories) and observed. Negative control sections were incubated in 1% BSA in PBS without lectin.
